# Estimating the determinants of health literacy for policy prioritisation: a local level case study in Newham, London

**DOI:** 10.1186/s12889-025-26067-9

**Published:** 2026-01-26

**Authors:** Nathan Green, Ysabella-Rozetta Hawkings

**Affiliations:** 1 Department of Statistical Science, UCL, London, UK; 2 Child Health Equity, Camden Council, London, UK

**Keywords:** MRP, Causal inference, Bayesian

## Abstract

This study investigates the determinants of health literacy in Newham, London, using a statistical modelling approach. Health literacy is defined using literacy, numeracy, and Information and Communication Technology (ICT) literacy from the ONS Skills for Life (SfL) survey 2011. The research combines survey, the Newham Residents Survey (NRS), and the UK 2021 Census data. Multilevel regression with post-stratification (MRP) and average treatment effects (ATE) are used to quantify the strength of association of various factors on health literacy at the local level.

The study identifies several significant determinants of health literacy, including age, ethnicity, qualification level, English as a first language, job status, gross income, and home ownership. Specifically, individuals aged 45 years and over are estimated to have lower ICT health literacy. White ethnicity is associated with higher numeracy scores. Additionally, health literacy worsens as area deprivation increases. Qualification level is estimated to be the strongest associated factor across all health literacy outcomes. The ultimate aim of this study is to inform targeted health literacy interventions at the local level by quantifying the relative importance of key determinants with uncertainty, thereby aiding in the prioritisation of resources.

**Supplementary Information:**

The online version contains supplementary material available at 10.1186/s12889-025-26067-9.

## Introduction

Health literacy is broadly defined as the ability to access, understand, appraise, and communicate health information, enabling individuals to engage in healthcare and maintain good health throughout their lives. Low health literacy is a significant concern and has received greater attention in recent years. It is associated with difficulties in making informed healthcare decisions, leading to poorer access to, knowledge, and quality of care.

Traditionally, health literacy was often assessed using unidimensional measures focused primarily on generic literacy and numeracy skills. However, there is a growing recognition of the multidimensional nature of health literacy, encompassing factors such as social support, navigation of healthcare systems, and communication skills. This broader perspective, as captured by tools like the influential Health Literacy Questionnaire (HLQ) [28], emphasizes the complex interplay of factors that influence an individual’s capacity to manage their health effectively.

Broadly, disparities in health outcomes have been linked to wider *social determinants of health (SDoH)*. SDoH are non-medical social, environmental and economic factors that have been shown to impact up to 90% of health status and disparities [42]. Health literacy itself is intricately related to SDoH, conceptualised in the literature in various ways: most commonly as a result of SDoH, but also as a mediator between other SDoH and health outcomes, or even as a modifiable in its own right [36].

Previous research has identified numerous socio-demographic factors consistently associated with lower levels of health literacy, such as lower educational attainment, older age, lower income, and racial/ethnic minority status [5, 18, 36]. Education, in particular, is frequently cited as the most important determinant of health literacy [13, 36]. Other associated factors include gender, marital status, depression, chronic conditions, and area deprivation [5, 15, 18, 36, 37]. Thinking has also shifted in recent years from it being wholly dependent on the individual to now recognise the role of the system and institutions in improving health literacy.

Research has shown a strong link between lower health literacy and poorer health outcomes, with studies using literacy and numeracy as proxies. A meta-analysis by [43] found that health literacy influences physical health, treatment adherence, and health behaviours, with lower levels linked to higher hospitalization rates, poor aftercare engagement, and negative attitudes toward seeking treatment.

There are several challenges and potential stigma associated with directly measuring health literacy in large populations or clinical settings, along with the time required [13, 18]. To address this, predictive models utilising available socio-demographic data have been explored to estimate health literacy levels and identify communities or individuals at higher risk [2, 13, 18]. Building on this previous work, this paper develops methods applied to social determinants of health and health literacy. We will adopt a statistical modelling approach with methods borrowed from causal inference and economics, and drawing on data from multiple sources.

This study focusses on Newham, a diverse borough in East London that faces unique challenges. Newham has been identified as having some of the lowest levels of health literacy in the UK [13, 41], a situation potentially compounded by factors such as a high prevalence of residents who speak English as a second language, its ranking as one of the most deprived boroughs in London, and the complexities of multigenerational or multiple-occupancy households.

To address this urgent need for better understanding, this study investigates the key determinants of health literacy in Newham. These findings can be used to produce rankings of the determinants in terms of their potential impact. Intervention prioritisation can target subpopulations to make best use of available resources and funds. The aim of this work is to investigate health literacy in Newham, using this borough as a case study to demonstrate a statistical methodology that can be applied by other local areas. This approach allows for the incorporation of associated uncertainties, an understanding of the relative importance of their own population characteristics, and can inform which health literacy interventions may be most appropriate and impactful for a specific local population.

## Methods

### Data

There are two types of data sets used in this study; Individual-level survey data to model associations between covariates and health literacy, and local-level data for the distribution of covariates in Newham. The latter comes from several different sources.

### Data for modelling associations

#### ONS Skills for Life (SfL) 2011 survey

The UK’s Office for National Statistics (ONS) Skills for Life (SfL) 2011 survey [1] was a comprehensive computer-based assessment conducted by the ONS to evaluate the skills of literacy, numeracy, and ICT (information and communication technology) of adults in England. The survey aimed to provide insights into the proficiency levels of adults aged 16-65 years old and to inform policy decisions and educational programs aimed at improving these essential skills.

The survey sampled 7230 participants in total, but it was designed to administer different assessments (particularly the more intensive ICT module) to pre-specified, planned subsets of the total sample. Multiple covariates were collected as part of the SfL survey. The survey data used in this work follows the analysis performed in [33] and we use the same set of covariates identified as significant. These include: age in years (16-44, $\ge$45), sex (male, female), ethnicity (white, non-white), job status (routine/manual/students/unemployed, intermediate, managerial/professional), working status (yes, no), UK born (yes, no), home ownership (yes, no), English as first language (yes, no), qualification level by General Certificate of Secondary Education (GCSE) (5 grade A–C GCSE or above, below 5 grade A–C GCSE), gross annual income (<£10,000, $\ge$£10,000) and Index of Multiple Deprivation (IMD). The survey provides raw IMD scores and groups according to the distributions of health literacy outcomes. We grouped the IMD data by quintiles to allow separation in the data because some of the group frequencies were very small. Other data collected that were not used included household composition, region of residence, occupation and industry sector, socioeconomic classification, self-reported health status, disability status.

#### Literacy, numeracy and ICT outcomes

The SfL survey tested the ability to read and understand text, calculate and manipulate numbers, extract and interpret information, and understand a range of vocabulary. They were also tasked with using a word processor, composing and sending emails, and using spreadsheets. Participants were graded according to the five lowest levels of the National Qualifications Framework (NQF) [40]. The NQF levels are EL1 and EL2 (Entry Level 1 and 2): These are a basic literacy level; EL3 (Entry Level 3): individuals at this level can understand and use simple texts; L1 (Level 1): This is considered the foundational level of literacy; L2 or above (Level 2 or above): This indicates a higher level of literacy proficiency. Individuals at Level 2 can deal with a wide range of written texts.

#### Data for local-level covariate distributions

To estimate the distribution of covariates specifically for Newham, we combined the following local-level data sources.

##### Newham Residents Survey (NRS)

The Newham Residents Survey (NRS) [27] is a periodic survey, usually every two years, conducted to gather detailed information on the views, experiences, and needs of residents in the London Borough of Newham. The survey covers various aspects of living in Newham, including satisfaction with local services, community safety, health and well-being, housing, and employment. This helps the local authority to make informed decisions and tailor services to better meet the needs of the community. Up until 2019, the survey was performed face to face, but due to COVID-19 in 2021 this switched to address-based online surveying, and the same survey method was used in 2023 due to cost and survey comparability. This means that 2021 is the baseline to monitor change. Random probability sampling is used at the community neighbourhood area level. Data are weighted using UK Census (2021) and Annual Population Survey (APS) data to ensure representativeness [24]. The weighting variables include age, gender, tenure, ethnic group, and working status. The APS is a major survey series, which aims to provide data that can produce reliable estimates at local authority level. Key topics covered in the survey include education, employment, health and ethnicity. Access to the individual-level data from the NRS allows us to directly estimate the joint distribution of covariates for Newham residents. We took age, sex, ethnicity, working status and home ownership from the NRS. The survey did not directly ask for gross individual annual income. Further, the only question about English language was about proficiency and so whether an individual had English as a first language could not be determined. As a result, we could not rely solely on using the NRS and needed to use other resources.

##### Additional data sources

For IMD data, the local income deprivation data set for mid 2021 population estimates for all 164 Lower Super Output Areas (LSOAs) in Newham was obtained from the ONS website [22]. This was then aggregated for each IMD decile population sum totals.

The Labour Force Survey (LFS) is a major household survey conducted by the ONS to gather essential statistics on employment, unemployment, and economic inactivity [23]. We used Quarterly Labour Force Survey microdata from July to September 2024 to estimate the joint distribution of the variables not in the NRS (highest level of qualification, English as a first language, UK born, job status and gross income). This was calculated using a type of iterative proportional fitting (IPF) (or *raking* in survey statistics) which is a method to match the marginal distributions (i.e. Newham) with the marginals from the microdata [4].

### Health literacy definition

Health literacy has several specific definitions [38]. We will adopt the definition used in [13] and [33], where health literacy is defined using the SfL survey variable for i) ICT skills (computer and internet use); ii) English literacy level (reading, writing, speaking and listening); iii) numeracy level (understanding and manipulating numbers).

In [33] a sample of health materials, including medicine labels, booklets, application forms and others were used which covered themes of health promotion, managing illness, systems navigation and disease prevention. All the materials were assessed for their literacy and numeracy complexity by education experts external. Education reviewers assessed the level of skill required to understand and use the materials. These were graded up to and including level 2, the level expected to be achieved by age 16 years; materials above this level were grouped with level 2.

SfL responses were mapped to the binary health literacy scale according to whether they are above or below the threshold determined by the education experts. The thresholds used were L2 for literacy, L1 for numeracy and EL3 for ICT.

#### Comparison of data sets

This section compares the demographic profiles of the SfL 2011 survey (our ‘source’ for modelling relationships) and local NRS 2023 dataset (our ‘target’ population). It is crucial to note that we are not comparing them to suggest they are similar. Rather, the purpose is to highlight any differences between the source and target data. This demographic divergence is the central methodological challenge that our MRP approach is designed to solve.

As shown in Table 1, there are stark demographic differences between source and target populations. The sample sizes were 5818 for literacy assessment, 4396 for numeracy assessment, and 2274 for ICT assessments. The total sample of the NRS 2023 consisted of 2270 valid records.

**Table 1:** Demographic and health literacy data summary table

		Skills for Life (SfL) 2011 Survey (Source Population)						Newham 2023 (Target Population)
		Literacy		Numeracy		ICT		
Variable	Category	*n*	%	*n*	%	*n*	%	%
Working Status	No	1859	32	1523	35	727	32	35
	Yes	3959	68	2873	65	1547	68	65
Gross Income (£)	<10000	796	14	636	14	285	13	10
	$\ge$10000	2298	39	1595	36	889	39	90
	Other	2724	47	2165	49	1100	48	-
UK Born	No	713	12	583	13	287	13	55
	Yes	5105	88	3813	87	1987	87	46
Sex	Female	3301	57	2582	59	1314	58	46
	Male	2517	43	1814	41	960	42	54
Own Home	No	2371	41	1966	45	932	41	65
	Yes	3447	59	2430	55	1342	59	35
Age (years)	16-44	3153	54	2394	54	1233	54	64
	$\ge$45	2665	46	2002	46	1041	46	36
English Language	No	476	8	395	9	175	8	35
	Yes	5342	92	4001	91	2099	92	65
Ethnicity	White	5191	89	3883	88	2044	90	30
	Non-white	627	11	513	12	230	10	70
Qualification	$\le$Level 1	1791	31	1555	35	694	31	43
	$\ge$Level 2	4027	69	2841	65	1580	69	57
IMD (decile)	1	7	0	9	0	4	0	2
	2	53	1	50	1	21	1	25
	3	132	2	111	3	52	2	45
	4	234	4	209	5	106	5	20
	5	495	9	431	10	184	8	6
	6	730	13	580	13	269	12	1
	7	1038	18	792	18	398	18	0
	8	1896	33	1370	31	752	33	0
	9	1233	21	844	19	488	21	0
Job Status	Lower	3035	52	2488	57	1168	51	56
	Intermediate	592	10	438	10	230	10	28
	Higher	2191	38	1470	33	876	39	17

Literacy, Numeracy and ICT are taken from the Skills for Life Survey 2011. The Newham population proportions are taken from the Newham Residents Survey 2023, unless otherwise stated. ONS Labour Force Survey 2024 data are used for English Language, Job Status and Gross Income. The ONS local income deprivation data set for mid-2021 was used for IMD

The population distribution is similar between the health literacy subsets of literacy, numeracy and ICT within the SfL survey data set but markedly different compared to the NRS. For example, UK born for SfL is 88% or 87% whereas for Newham this is 46%. This clearly corresponds to English as first language and White ethnicity being lower for Newham at 65% (91-2% for SfL), and 30% (88-90% for SfL), respectively. IMD for Newham is concentrated in deciles 2, 3 and 4 with only a few or no counts in the others.

**Fig. 1:**
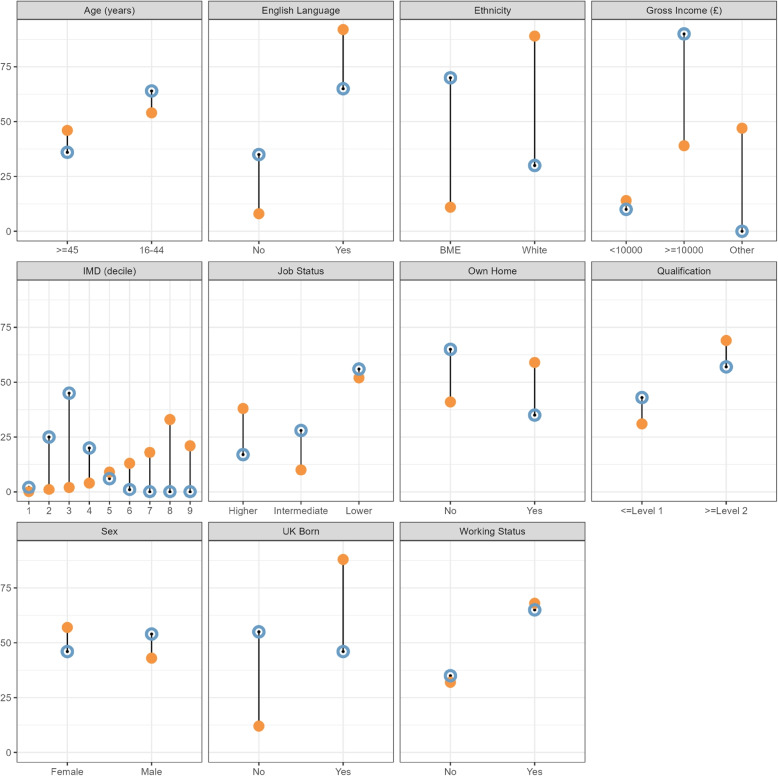
Dumbbell plots of demographics proportions for the Skills for Life survey 2011 (solid orange) and Newham Residents Survey 2023 (hollow blue) by variable and category. The solid lines indicate the difference between proportions

This significant demographic divergence is a central methodological challenge this study addresses. It confirms that simple estimates from the national SfL survey cannot be directly applied to a unique local area like Newham. Figure 1 shows dumbbell plots of the differences between SfL 2011 data set and Newham Residents Survey 2023 for the literacy outcome. Other outcome plots are given in the Appendix.

#### Sensitivity analysis

To test the robustness of our findings and address the limitation of using the 2011 SfL survey, we conducted a sensitivity analysis by replicating our MRP-ATE models using two alternative datasets: the 2003 Skills for Life (SfL) survey and the most recent UK Programme for the International Assessment of Adult Competencies (PIAAC, Cycle 2) 2023 data (from the Organisation for Economic Co-operation and Development (OECD)).

However, these datasets presented significant limitations for direct comparison. The 2003 SfL survey, while using comparable outcomes, is coded differently to SfL 2011 and does not contain all of the same covariates. It also precedes the widespread adoption of digital health technologies. The 2023 PIAAC data contains only two of our three outcomes (omitting ICT) and is missing many key covariates used in our primary model (e.g. job status, home ownership). Furthermore, crucially, the PIAAC outcome scales are different from the SfL scales. They required an approximate mapping (taken from an analysis by the UK Department for Business, Innovation and Skills (BIS)) to the health literacy thresholds defined by [33], introducing additional uncertainty. The BIS report [1] explicitly states that, although the sets of assessments in SfL and PIAAC have similar objectives, they are constructed differently, have different assessment criteria, weightings and test different skills.

Despite these limitations, we proceeded with the analysis to check if the main determinants remained consistent. Where covariate data were missing we imputed values using multiple imputation, which introduced further uncertainty in the model. The full methodology and comparative results are presented in the Appendix.

### Statistical analysis

Relevant information is available from different datasets, but each has limitations for our research question. The NRS is current and specific to Newham but lacks health literacy data. The SfL survey, conversely, provides literacy, numeracy and ICT measures that can be transformed into health literacy outcomes using Rowland *et al*. [33]. The SfL survey is not specific to Newham but is from a subset of a different population. Our central methodology is designed to address the challenge of having no single dataset that contains both current, local-level demographics and health literacy outcomes. We do this not by merging datasets at the individual level. Instead, we use the two-stage process of *Multilevel Regression with Post-Stratification (MRP)*.

#### Multilevel Regression with Post-Stratification (MRP)

MRP is a statistical technique that combines multilevel modelling with post-stratification to estimate subnational characteristics from survey data [29, 30]. It accounts for hierarchical data structures (e.g. individuals within groups) and adjusts for known population characteristics, yielding more precise small-area estimates. MRP allows inference on a population of interest from sparse or non-representative samples, integrating small-area estimation and population adjustment techniques. Although used in political science [10, 14], its public health applications are relatively few. MRP proceeds in the following two stages.

**Multilevel Regression**: A multilevel model of individual survey responses (from SfL data) is estimated to generate an estimate for each demographic group. Our model uses a binary outcome $y_i$ for individual *i* (above/below health literacy threshold). The predicted probability $\hat{\pi }_i$ is defined as:

$$\begin{aligned} \hat{\pi }_i = \text {logit}^{-1} \left( \hat{\beta }_0 + \sum \limits _{x} \hat{\beta }^{x}_{\gamma _x[i]} \right) \end{aligned}$$

where $\hat{\beta }_0$ is the intercept, $\hat{\beta }^{x}_{\gamma _x[i]}$ are coefficients for covariates *x* (age, sex, eng, white, ukborn, qual, inc, job, work, home), and $\gamma _x[i]$ represents the level or category for covariate *x* for individual *i*. IMD is included as multilevel random effects $\beta ^{\text {IMD}}_j \sim \text {N}(\mu _{\text {IMD}}, \sigma _{\text {IMD}}^2)$. Priors distributions for fixed effects are normal distributions centered at zero with modest variance, and half-normal priors are used for random effect standard deviations [8].

**Post-Stratification**: The health literacy probabilities for each demographic category (cell *c*) are weighted by their proportion in the actual Newham population. With 11 covariates resulting in $|\mathcal {S}|$ = 13,824 cells, the post-stratified estimate $\hat{\pi }^{\text {mrp}}$ is:

$$\begin{aligned} \hat{\pi }^{\text {mrp}} = \sum \limits _{c = 1}^{|\mathcal {S}|} w_c \hat{\pi }_{c} \end{aligned}$$

where $\mathcal {S}$ is the set of all covariate combinations, $N_c$ is the population frequency for cell *c*, *N* is the total population size, and $w_c = N_{c} / N$ are the combination weights.

#### Predictive comparisons and MRP

The *Average Marginal Effect (AME)* is commonly used to interpret regression results on the outcome scale, measuring the change in outcome probability due to an incremental change in a predictor [20, 25, 26]. We adopt this idea but from an Average Treatment Effect (ATE) perspective, since they can be thought of as equivalent for a binary valued treatment covariate. This approach is related to causal estimation but we adopt the term *predictive comparison* from [9] to emphasize that we are summarizing the structure of the predictive model and not necessarily estimating causal effects.

We then use MRP to estimate target population specific effects with an ATE model, which we shall call *MRP-ATE*. This is an area explored by Gao [7]. Under the potential outcome framework [11], the ATE on the population level is ${\mathbb {E}[Y(1) - Y(0)]}$, which averages the effect of an intervention (e.g. changing a covariate from 0 to 1) across the entire population. We define the MRP-ATE for a particular target population (different from the sample population) as

$$\begin{aligned} \text {MRP-ATE} & \approx \sum \limits _{x \in \mathcal {S}} ( \mathbb {E}[\pi \mid T=1, X = x ] - \mathbb {E}[\pi \mid T=0, X = x ] ) w_x \\ & = \hat{\pi }^{\text {mrp}}_{T=1} - \hat{\pi }^{\text {mrp}}_{T=0} \end{aligned}$$

This allows us to investigate the impact of changing a covariate’s value (our “treatment”) on health literacy, even for non-modifiable factors like age or sex. For example, we can model the effect of an intervention that makes older individuals’ ICT health literacy “as-if” they were younger. We assume that the chosen covariates are appropriate confounders across all focal covariates.

#### Priority ranking

For policy applications such as healthcare cost-effectiveness, it’s useful to rank different options. We work with the *cumulative rank probability* ($P_{ir} = \sum \nolimits _{k=1}^{r }p(R_i = k)$), which indicates the total probability of a variable achieving a given rank or higher. A key feature of our analysis is using the *absolute treatment effect*, $|Y(1) - Y(0)|$, for ranking, acknowledging that the direction of the effect can be arbitrary in model choice. To summarize these probabilistic rankings, we adopt the *Surface Under the Cumulative Ranking Curve (SUCRA)* metric, common in multiple-treatment meta-analysis [34]. SUCRA represents the percentage of the maximum possible cumulative rank an intervention (in our case, an input variable) can achieve, providing a single value where a higher SUCRA indicates a better overall rank relative to others. For our model, it is given by the following

$$\begin{aligned} \text {SUCRA}_{ij} = \sum \limits _{r=1}^{n-1} P_{ijr} / (n-1), \end{aligned}$$

where $P_{ijr}$ is the cumulative probability for variable *i* at level *j* and rank *r*. The mean rank is

$$\begin{aligned} \mathbb {E}[\text {rank}(i,j)] = n - \sum \limits _{r=1}^{n-1} P_{ijr}. \end{aligned}$$

#### Software implementation

All data preparation and analyses were performed with the statistical programming language R v.4.5.0 [32] calling Stan for Bayesian analyses. Uninformative prior distributions were used for all parameters and posterior samples obtained. Code is available on GitHub at https://github.com/n8thangreen/healthliteracy. The MRP code is adapted from the code provided in [3]. IPF was performed with the *simPop* package in R [39].

## Results

**Fig. 2:**
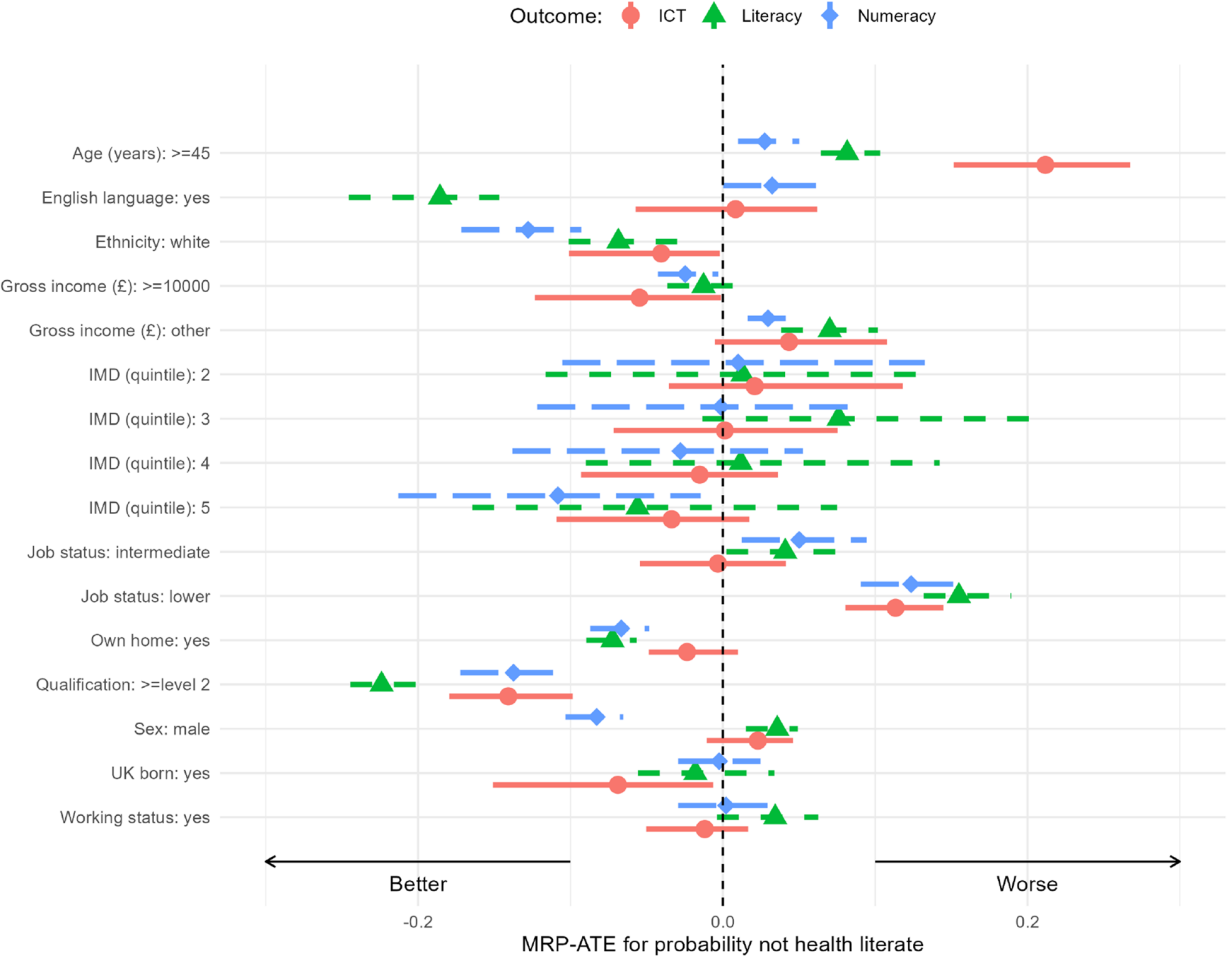
Forest plot of posterior distributions of MRP-ATE for probability of not health literate for each of the outcomes ICT (red circles), literacy (green triangles) and numeracy (blue squares)

Figure 2 shows a forest plot of the MRP-ATE for the probability of low health literacy, broken down by each outcome. The plot displays the posterior distribution means and their 95% credible intervals. The labels on the *y*-axis indicate a particular covariate level. The corresponding data point shows the estimated outcome if all individuals in the Newham population were assigned to that level, while averaging over all other covariates. Negative values demonstrate an improvement in health literacy and positive values a worsening. The corresponding values are given in Table 2.

**Table 2:** Multilevel regression with poststratification average treatment effects (MRP-ATE) for each variable by probability not health literate outcomes ICT, literacy and numeracy

Variable/Category	Literacy	Numeracy	ICT
Age (years)			
$\ge$45	0.082 [0.064, 0.103]	0.027 [0.010, 0.050]	0.212 [0.152, 0.267]
English Language			
Yes	-0.186 [-0.246, -0.147]	0.032 [0.000, 0.071]	0.008 [-0.057, 0.062]
Ethnicity			
White	-0.069 [-0.101, -0.030]	-0.128 [-0.172, -0.093]	-0.041 [-0.101, -0.002]
Gross Income (£)			
$\ge$10000	-0.013 [-0.036, 0.010]	-0.025 [-0.043, -0.003]	-0.055 [-0.123, -0.001]
Other	0.070 [0.038, 0.102]	0.030 [0.016, 0.051]	0.043 [-0.005, 0.108]
IMD (quintile)			
2	0.013 [-0.116, 0.136]	0.010 [-0.105, 0.133]	0.021 [-0.035, 0.118]
3	0.076 [-0.013, 0.204]	-0.001 [-0.122, 0.090]	0.001 [-0.072, 0.075]
4	0.012 [-0.090, 0.142]	-0.028 [-0.138, 0.053]	-0.015 [-0.093, 0.036]
5	-0.056 [-0.164, 0.075]	-0.108 [-0.213, -0.015]	-0.034 [-0.109, 0.017]
Job Status			
Intermediate	0.041 [0.002, 0.081]	0.050 [0.012, 0.094]	-0.003 [-0.054, 0.041]
Lower	0.155 [0.132, 0.189]	0.124 [0.090, 0.159]	0.113 [0.080, 0.145]
Own home			
Yes	-0.072 [-0.090, -0.056]	-0.067 [-0.087, -0.048]	-0.024 [-0.049, 0.010]
Qualification			
$\ge$level 2	-0.224 [-0.245, -0.190]	-0.137 [-0.172, -0.106]	-0.141 [-0.180, -0.099]
Sex			
Male	0.036 [0.015, 0.049]	-0.083 [-0.103, -0.065]	0.023 [-0.011, 0.046]
UK Born			
Yes	-0.018 [-0.056, 0.034]	-0.002 [-0.029, 0.025]	-0.069 [-0.151, -0.006]
Working Status			
Yes	0.034 [-0.004, 0.063]	0.002 [-0.029, 0.029]	-0.012 [-0.050, 0.017]

Mean change in probability of not being health literate is given, with upper and lower bounds of the 95% intervals

The plot clearly shows that the ICT outcome is not associated as significantly as literacy and numeracy when changes in covariate values occur. The exception to this is age where people over 45 years old have a markedly worse change in ICT scores compared to literacy and numeracy scores (0.192 [0.146, 0.258]). Numeracy scores are associated most by a change to White ethnicity and qualification level being level 2 or higher (-0.129 [-0.160, -0.095] and -0.141 [-0.166, -0.117] respectively). All outcomes (ICT, literacy, and numeracy) were associated positively by having a qualification at level 2 or above - the only factor to have a wholly positive association across all three outcomes. The ICT outcome tends to have smaller uncertainty for IMD and no improvement for less deprived areas compared to the other outcomes. For literacy and numeracy, less deprived areas indicate improvement but are clearly not significant. ICT and in particular literacy have worse health literacy estimates for more deprived quintiles relative to quintile 1 (most deprived). The IMD results exhibit borrowed strength and shrinkage to the pooled mean due to the Bayesian multilevel property which regularised the fit. Recall, there are few Newham residents in quintile 1 thus pulling it more towards the pooled mean value and most of the residents are in quintiles 2 and 3. Counter-intuitively, quintile 3 for literacy appears to actually be worse than quintile 1. This is likely an artifact of the Bayesian multilevel model, as Newham has few residents in quintile 1. A simpler model with a fixed effect for IMD gives an OR of 0.74, indicating a consistent improvement for less deprived areas. This finding corresponds with the original [33] analysis. Male sex shows an increase in numeracy (-0.092 [-0.119, -0.066]). Unsurprisingly, English as a first language has a strong benefit for literacy (-0.171 [-0.217, -0.130]) but little to no influence on the other health literacy outcomes.

**Fig. 3:**
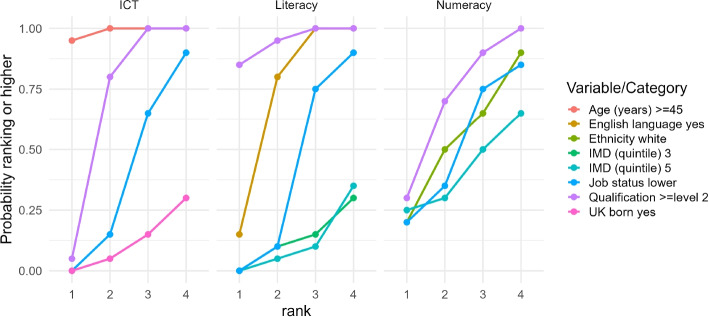
Rankogram plots of cumulative rank probabilities for the first four positions and each of the outcomes of not health literate for ICT, literacy and numeracy. Only probabilities above 0.25 are shown for clarity. MRP-ATE for absolute treatment effects are used to rank so magnitude and not direction of effect relative to reference category is indicated

Figure 3 provides a different view of the results useful for decision-making, showing rankogram plots of cumulative ranks probabilities $P_{ijr}$. These plots show the likelihood of a variable being ranked 1st to 4th as the variable which will have the largest association on the outcome (ICT, literacy, or numeracy) in terms of change in MRP-ATE. The strength of this plot is that it allows us to make direct comparison between the different covariates accounting for both the effect size and uncertainty. We restrict the plot to only show the variables with a minimum cumulative rank of at least 0.25 so that the plot is clearer and focuses on the most important variables. Recall that the absolute treatment effects are used to rank so the magnitude but not direction of the MRP-ATE relative to reference category is indicated. The full rankogram plot with all categories and all ranks is given in the Appendix Fig. 4.

For ICT, the $\ge$45 age threshold clearly has the highest chance of ranking in the top positions. The next two best ranking variables are clearly qualification then lower job status. For literacy, qualification has the highest probability of ranking first. English as first language has the next highest probability of ranking second. Finally, for numeracy, it is less clear cut which variable is the top ranking. Qualification is the top ranking but with less certainty with both white ethnicity and lower job status also competing. A lack of deprivation, i.e. upper IMD quintile, is also not insignificant.

Similar to the rankogram plot, Table 3 shows the SUCRA and expected rank statistics for each variable and health literacy outcome using the absolute value of the MRP-ATE estimates. The values correspond between the two statistics since they are both derived from the cumulative rank. Qualification greater than level 2 ranks first or second across all outcomes. English language ranks second for literacy, and age $\ge$45 years old ranks first for ICT.

**Table 3:** SUCRA and expected rank using absolute value MRP-ATE for the health literacy outcomes ICT, literacy and numeracy

		SUCRA			$\mathbb {E}$[rank]		
Variable	Category	ICT	Literacy	Numeracy	ICT	Literacy	Numeracy
Age (years)	$\ge$45	100	66	27	1	6	12
English Language	Yes	33	93	34	11	2	11
Ethnicity	White	46	56	88	9	8	3
Gross Income (£)	$\ge$10000	55	11	28	8	14	12
	Other	49	55	32	9	8	11
IMD (quintile)	2	34	35	47	11	11	9
	3	36	51	40	11	8	10
	4	39	40	45	10	10	9
	5	43	49	74	10	9	5
Job Status	Intermediate	31	30	49	11	12	9
	Lower	85	85	87	3	3	3
Own Home	Yes	36	58	62	11	7	7
Qualification	$\ge$Level 2	92	99	93	2	1	2
Sex	Male	32	28	70	11	12	5
UK Born	Yes	63	16	13	6	14	14
Working Status	Yes	26	26	11	12	12	14

## Discussion

We estimated an increase in numeracy health literacy for UK born. This may be due to non-UK born may have received their primary and secondary education in different countries with varying curricula and teaching standards, which could affect their performance on a UK-based skills assessment [21]. Broader societal issues, such as systemic biases within the education system or other institutions, can create disadvantages for ethnic minority groups. These disadvantages can manifest as disparities in access to high-quality education, resources, and support, which in turn affect the development of skills such as numeracy.

The health materials used to set the competency thresholds were assessed for both their complexity in literacy and numeracy. Numeracy tests, especially those based on real-world scenarios such as health information, are not just about pure calculation. They often involve word problems that require strong reading comprehension to understand the context and what is being asked. Therefore, lower English literacy skills could directly hinder performance on a numeracy test, even if the person’s mathematical ability is strong [12]. Finally, it may be that societal factors and stereotypes can sometimes create higher levels of “maths anxiety” in certain demographic groups [16]. This can affect confidence and performance in testing situations specifically related to mathematics and numeracy, independent of other academic skills.

The decrease in health literacy for literacy may be a spurious result for this data set or it may indicate something about a focus of government funding, non-profit aid, and specific support programs on the most deprived areas and the next most deprived may be falling between the cracks.

We also recognise that the ethnicity categorisation as taken from the SfL survey is overly simplistic (originally coded White or Black and Minority Ethnic (BME) only). This is an increasingly outdated approach that masks significant diversity and is a significant limitation for diverse populations in areas such as Newham [35].

We showed that a more deprived area tends to correlate with a decrease in health literacy outcomes. This corresponds with other more specific variables since IMD can be thought of as a compound measure. IMD includes a domain for education, skills, and training deprivation. Less deprived areas scores often have lower levels of educational attainment. We overwhelmingly identify qualification level as the strongest associated factor across all health literacy outcomes. Therefore, the educational disadvantage captured by IMD directly translates to lower literacy and numeracy skills, which are the core components of the health literacy definition used in this research. Limited access to resources includes less access to computers and the internet; reduced ability to afford stable housing, adequate or high quality nutrition, and other resources that support overall well-being as well as the capacity to engage with health services potentially due to limited time and energy to focus on preventive health or decipher complex health information due to the stresses of financial instability and day-to-day challenges and pressures [17]. It is also recognized that people experiencing social complexity and systemic inequality may have reduced trust in institutions. This is often because those systems have failed to support them previously . This reduced trust can, in turn, further affect their access and capacity to engage with health services [19].

The University of Southampton and NHS England created a web tool to estimate the percentage of a local authority population with low health literacy and numeracy. It also used the 2011 Skills for Life Survey with a population update based on the 2021 Census and the 2019 Index of Deprivation [41]. Their estimated health literacy prevalence in Newham was 74.78% (second lowest in England). Newham’s neighbouring boroughs included Barking and Dagenham 78.41%, Tower Hamlets 67.61% and Greenwich 66.35%. The national average was 58.30%. These boroughs are somewhat similar to Newham and its population’s demographics, with Tower Hamlets being the most comparable.

### Sensitivity analysis

The results from our sensitivity analysis confirmed that our main conclusions are robust. While the exact magnitude of the ATEs differed, as expected given the different datasets and covariate limitations, the relative importance of the key determinants remained consistent.

Specifically, qualification level was identified as the most important factor to consider across all three analyses (SfL 2003, SfL 2011, and PIAAC 2023). Similarly, age remained the strongest predictor for ICT literacy in the 2011 data (although it was not available in PIAAC 2023), and English as a first language remained a key predictor for literacy in all datasets. This gives us confidence that the determinants identified in our primary analysis are not an artifact of the 2011 data but reflect persistent underlying relationships.

## Conclusions

This study provides an extensive, qualitative analysis of the determinants of health literacy in Newham, London, using advanced statistical techniques and multiple data sets. The research identifies several key factors that are significantly associated health literacy outcomes, with implications for targeted interventions and policy-making in the area.

Health literacy in this study is defined using the Skills for Life (SfL) 2011 survey, encompassing literacy, numeracy, and ICT skills. This definition allows for a comprehensive assessment of health literacy, moving beyond traditional unidimensional measures. The analysis combines data from the SfL and LFS surveys, the Newham Residents Survey (NRS), and the UK 2021 Census. The NRS and Census data provide local demographic information. This combination of data sets addresses the limitations of each individual source, allowing a more complete understanding of the factors that influence health literacy in Newham.

We employed multilevel regression with post-stratification (MRP) to estimate health literacy at the subnational level, to provide more precise estimates for small areas and subgroups. The research uses average treatment effects (ATE) to quantify the strength of association of various factors on health literacy, providing insights into how changes in these variables affect health literacy outcomes. Key determinants of health literacy were identified by [33], including age, ethnicity, qualification level, English as a first language, job status, gross income, and home ownership. These factors have varying impacts on different aspects of health literacy.

A motivation for ranking the determinants of health literacy was to facilitate communication with public health professionals. By translating complex statistical outputs into a more intuitive format, ranking helps to clearly identify which socio-demographic factors have the most substantial size of association on literacy, numeracy, and ICT skills within Newham. This prioritisation is crucial for effectively allocating limited resources. However, ranking does simplify multifaceted issues and the relative importance of factors can be sensitive to the specific statistical model and data used. Therefore, rankings can be a powerful communication tool, but are only a guide.

Health literacy interventions at the individual level include, educational programs, simplified health communication strategies, digital health tools, and numeracy training to improve comprehension and decision-making. Healthcare system-level interventions include training providers in clear communication, screening for low health literacy, offering patient navigation services, and ensuring culturally and linguistically appropriate materials. Community-based and policy interventions involve integrating health literacy into schools and workplaces, public health campaigns, partnerships with local organizations, and advocating for policies that promote equitable access to health information. These strategies aim to empower individuals and improve health outcomes.

With new plans for the NHS being published recently, the need to pivot the English health system to a prevention-first approach, improving health literacy of institutions and individuals is a key opportunity and underpinning factor that will need to be addressed to improve access, knowledge, and experience for patients, staff, and the public. This data and any future tool created will help the stakeholders in a local area to understand the factors that largest association with health literacy, and therefore enable them to consider how they can adjust their practice or deliver an intervention which can improve health literacy for the residents they work with. At a time when local authority budgets and resources are stretched and limited or being reduced, being able to focus the limited resources on interventions more likely to have an impact supports best practice for effective and efficient use of public funds. The results of this work can inform targeted health literacy interventions in Newham, focussing on the groups and areas with the greatest impact. An up-to-date quantification can better guide resource allocation and policy decisions, ensuring that interventions are tailored to the specific needs of the community.

There are several key directions for future research and practice.

Building on previous work, such as the Geodata app [41], future work should look to develop an accessible, interactive web tool with the methods presented here, such as a Shiny app, to allow stakeholders to explore their own data and context dynamically, enabling a more detailed, evidence-based approach to developing new health literacy interventions.

Alternative or additional existing data sets, such as those available from the ONS Understanding Society UK Household Longitudinal Study or PIAAC may be considered in the future. The UK PIAAC survey does in fact collect data for many other covariates which are not in the public release. Future extracts may be more appropriate for our analysis.

Further, as local authorities increase the coproduction and data collection they undertake both internally and with residents, there are opportunities to collect additional local data which will provide more locally accurate and up-to-date data, e.g. the Newham Residents Survey. This could be used to validate the methods and extended to provide the full covariate correlation structure required for MRP. To address not currently have access to this, we employed iterative proportional fitting (IPF) in order to synthetically simulate the required data using marginal summary statistics. Alternative methods are available and this is an area of active research [31].

It will be necessary to align with best practice work which is developed nationally, regionally, and locally to ensure the model iterates to incorporate the data which can evidence the impact of best practice locally. Therefore, it would be beneficial to consider measures which show change quickly (e.g. annual data collection) that could be included in the model to enable close to real-time monitoring of interventions to evidence impact and inform iterations.

In addition, more granular data (e.g. ethnicity) and more culturally appropriate or sensitive data collection methods will provide additional insights to inform community-specific approaches and provide a more accurate understanding of how standardised measurements such as the SfL may affect the measures of ICT, literacy, and numeracy (e.g. English comprehension being required to complete mathematics questions). This will also help institutions to assess where different approaches may be necessary for different groups of patients.

The ATE estimates, as used in our analysis, provided the largest change on the outcome scale between two discrete category counterfactual scenarios. This is natural in terms of a rate of change or gradient, as in an average marginal effect model. For our context, an alternative approach could be to use the *average treatment effect on treated (ATT)*. This is the ATE for only the subset of population that would have their covariate value changed and does not include those who already have that value. Further, there exist ATE variants for heterogenous, marginal and quantile treatment effects. Finally, additional modifications can be made to account for relative population sizes, importance or budgets.

We also assumed that it is possible to intervene on a covariate without affecting the remainder. It may be that a direct change to one covariate has an effect on the other covariates which in turn has an indirect effect on the outcome [6]. It may also be that possible values available for an intervention for an individual may depend on their particular set of covariates. Finally, we have assumed deterministic interventions but this may be generalised to *stochastic* interventions, loosely defined as interventions that yield a random variable focal covariate.

We acknowledge that the use in our primary analysis of the 2011 SfL survey is a key limitation. However, we retained this as our main data set because it is the sole data set that contains all three of our specified outcomes (literacy, numeracy, and ICT) and, most importantly, is the source data used by [33] to create the validated mapping of skills to real-world health materials that defines our health literacy outcomes. To mitigate this limitation, we conducted a sensitivity analysis using the 2023 PIAAC and SfL 2003 data. As detailed in the Appendix, this analysis confirmed that our central conclusions, particularly the overwhelming importance of qualification level, remain robust. This suggests that while the prevalence of skills may have changed, the socio-demographic determinants of those skills have remained relatively consistent.

Up-to-date, local, detailed data should be collected to best inform specific questions. In turn, all available evidence should be principally synthesised if relevant even indirectly; a problem suited to a Bayesian paradigm. For example, the current analysis could be extended to include multiple surveys over time or location to capture temporal and spatial variation and allow future predictions. Further, research is needed to quantify the cost-effectiveness of health literacy initiatives and to rigorously evaluate their long-term impact on clinical and health outcomes.

There is an ongoing need for more sensitive and comprehensive measures of health literacy, particularly for diverse populations such as Newham and within evolving digital health environments. This includes developing methods to accurately assess digital health literacy among digitally marginalized groups. For example, development of innovative tools, such as AI-driven text simplification or translation, could make health information more accessible. However, rigorous evaluation is necessary to ensure accuracy and comprehensibility of such technologies.

Health literacy, and any interventions aim at addressing it, are closely linked to health patient activation. This refers to an individual’s confidence and motivation to manage their own health and healthcare. The Patient Activation Measure (PAM) is a common validated tool that assesses this, ranging from passive and overwhelmed to proactive and confident. It can be used as a basis to develop interventions targeted at communities most of risk of missing out from likely gains in digital health such as the NHS app.

Our work will help inform targeted and tailored interventions to prioritize the design and implementation of community-based, culturally appropriate interventions. Incorporating health literacy screening into standard healthcare practices can help identify patients needing additional support and facilitate timely provider responses. However, it should be noted that the potential for driving inequalities and inequities will need to be mitigated against (e.g. preventing biases and stigma due screening outcomes). Furthermore, in populations with low trust in the ‘system’ much of the data used in the model may not be available on every patient record which would limit the accuracy and effectiveness of screening tools that rely on demographic data and the like, especially those not regularly collected or updated (e.g. employment) in health and care records.

Alongside individual health literacy development, there must be a sustained focus on improving *organizational* health literacy. This involves ensuring that healthcare systems and public health organisations are designed to provide clear, accessible and culturally and linguistically appropriate health information and services in all communications (including face-to-face or verbal sharing of information). As new determinants of health emerge and become more understood, such as digital and commercial determinants, continued research and policy attention are required to understand their association on health literacy and to develop proactive strategies to mitigate potential inequities.

## Supplementary Information

Supplementary Material 1.

## Figures and Tables

**Fig. 4:**
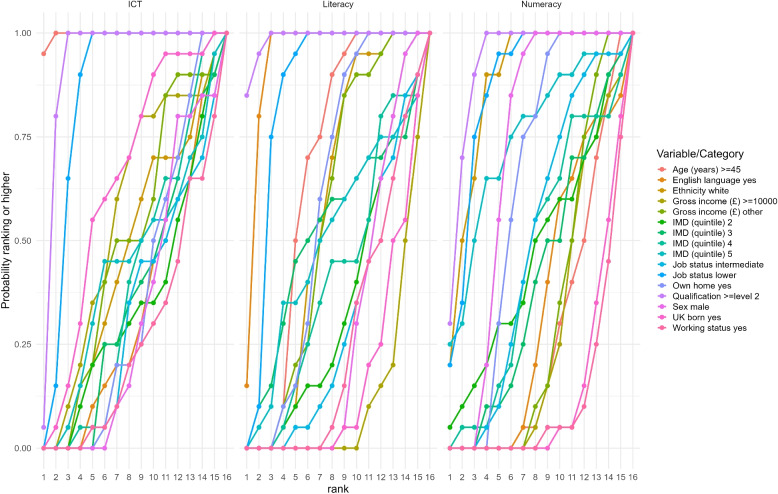
Cumulative rank probability plots for absolute MRP-ATE outcomes for all ranks and all variable categories

**Fig. 5:**
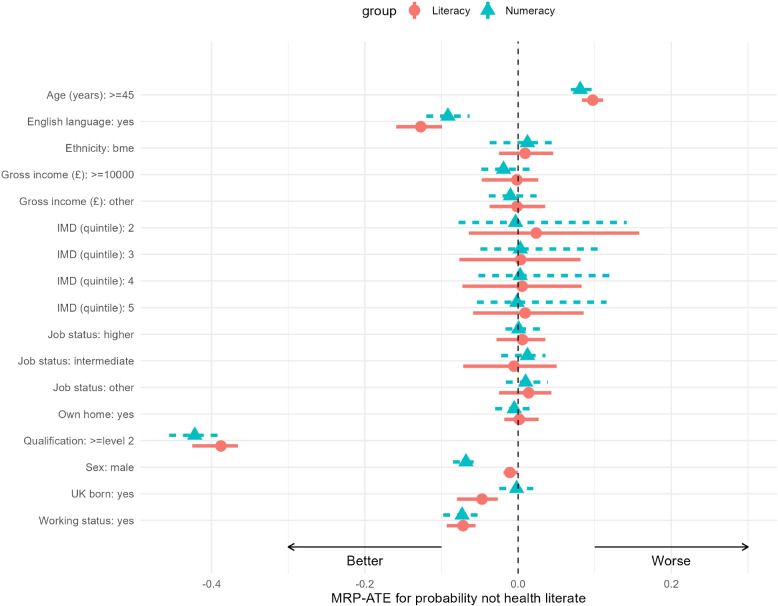
Forest plot of posterior distributions of MRP-ATE for probability of not health literate using PIAAC 2023 data with imputation, for outcomes literacy (red circles) and numeracy (blue triangles)

**Fig. 6:**
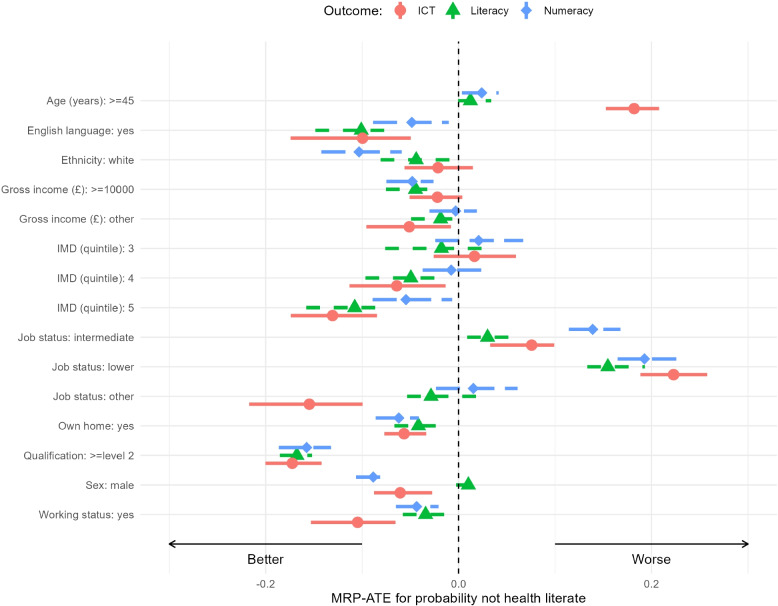
Forest plot of posterior distributions of MRP-ATE for probability of not health literate using Skills for Life 2003 survey for each of the outcomes ICT (red circles), literacy (green triangles) and numeracy (blue squares)

## Data Availability

Skills for Life 2011 dataset is available at https://datacatalogue.ukdataservice.ac.uk/studies/study/7240#details. Skills for Life 2003 dataset is available at https://datacatalogue.ukdataservice.ac.uk/studies/study/7239#details. PIAAC data is available at https://www.oecd.org/en/about/programmes/piaac/piaac-data.html. The dataset on Labour Force Survey is available at UK Data Service https://datacatalogue.ukdataservice.ac.uk/series/series/2000026#abstract. Newham Resident Survey dataset is held by the London Borough of Newham which were used under license for the current study, and so are not publicly available. UK Census data are available from https://www.ons.gov.uk/census. Data sets generated during the current study are available from the corresponding author on reasonable request.

## Appendix

The cumulative rank probability plot for all variables with SfL 2011 data is given in Fig. 4.

## Sensitivity analysis

The PIAAC 2023 data set was missing IMD, ethnicity, gross income, job status and own home. In order to use the same model and make a comaprision, these covariate values were imputed using multiple imputation. The observed data from SfL 2011 were used to estimate the missing covariates. The R packages mice and brms were used to perform this. Twenty imputation samples and 20 iterations were used in the algorithm.

Similarly, the Skills for Life 2003 survey was missing a smaller number of covariates (UK Born and English as first language) and had different codings for job status. The analysis was run on the available matching covariates to ensure comparability with the primary 2011 model

Figures 5 and 6 show the MRP-ATE forest plots using the PIAAC 2023 data with imputation and Skills for Life 2003 survey data, respectively.

Despite the differences in data, a comparison of these plots with our primary analysis confirms our main conclusion. In both the 2003 and 2023 analyses, qualification level remains the strongest and most consistent factor associated with both literacy and numeracy. This provides strong validation that our primary finding is robust over time. A full summary of these validation results is provided in the main Discussion section.
