# Supplementary figures and images for: Estimating the determinants of health literacy for policy prioritisation: a local level case study in Newham, London
Source: BMC Public Health. 2026 Jan 26;26:649. doi: 10.1186/s12889-025-26067-9 (PMC12918084; doi:10.1186/s12889-025-26067-9)

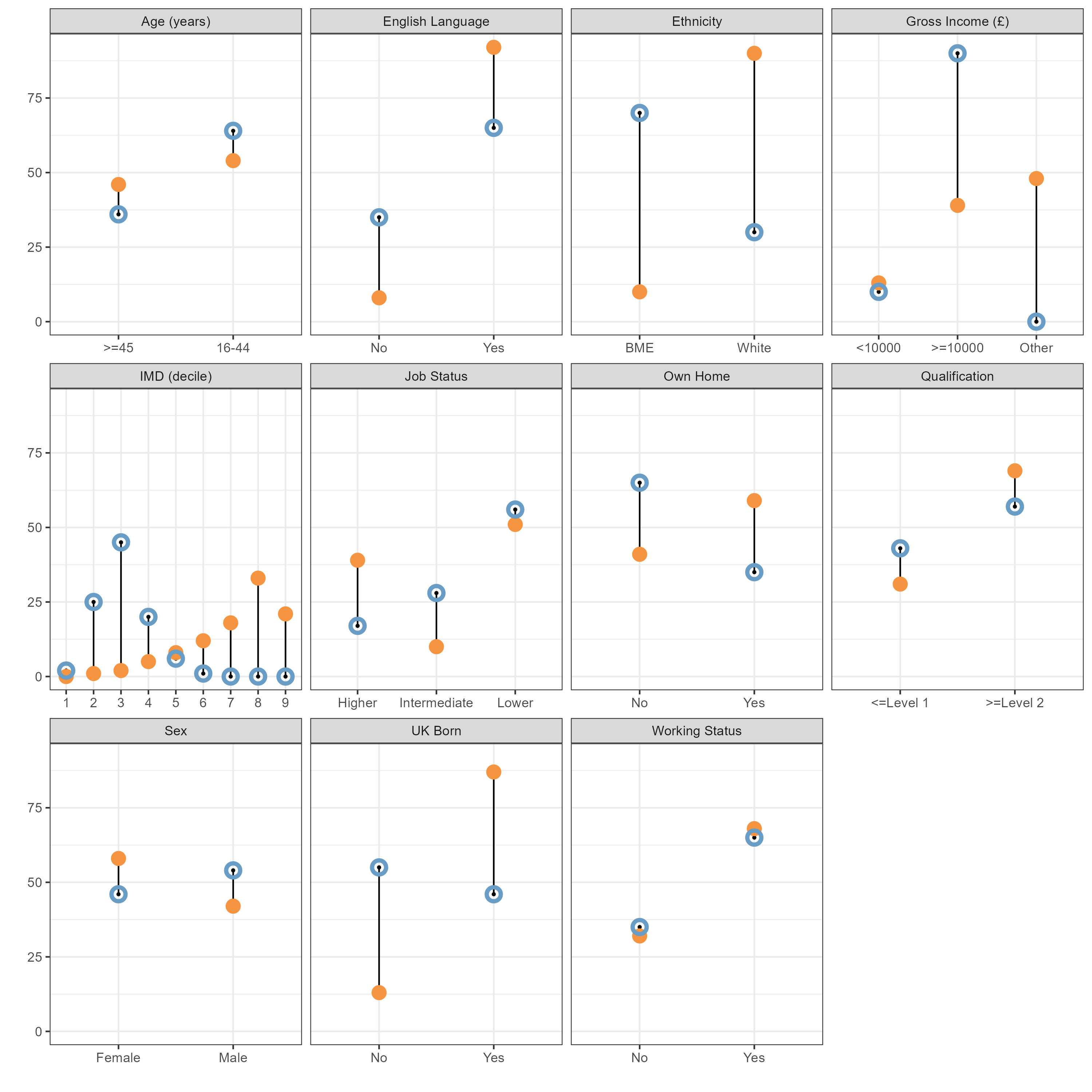

Supplement: Supplementary file 1 — Supplementary Material 1. [file 12889_2025_26067_MOESM1_ESM.zip › dumbbell_plot_ICT.png]

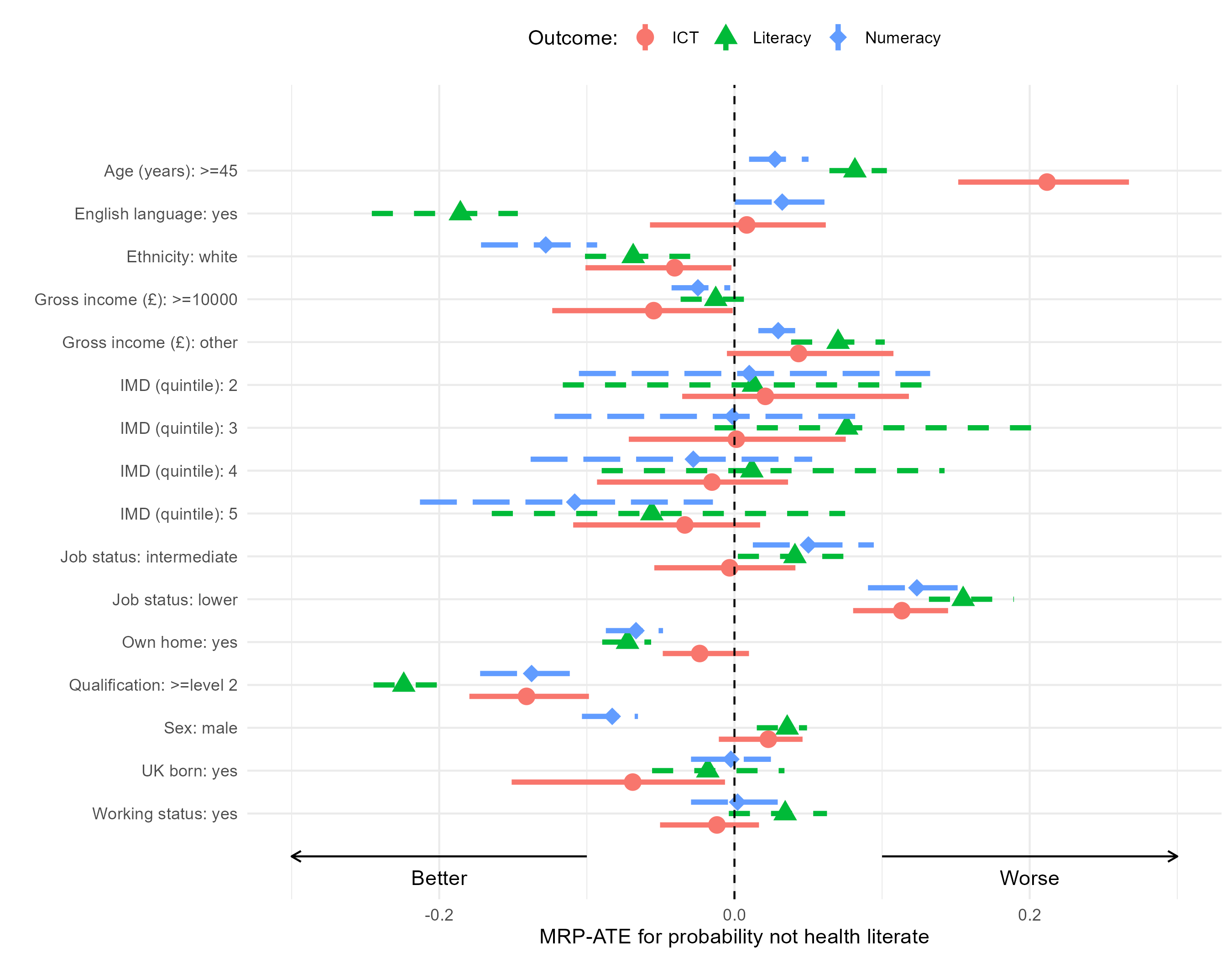

Supplement: Supplementary file 1 — Supplementary Material 1. [file 12889_2025_26067_MOESM1_ESM.zip › ame_forest_group_plot.png]

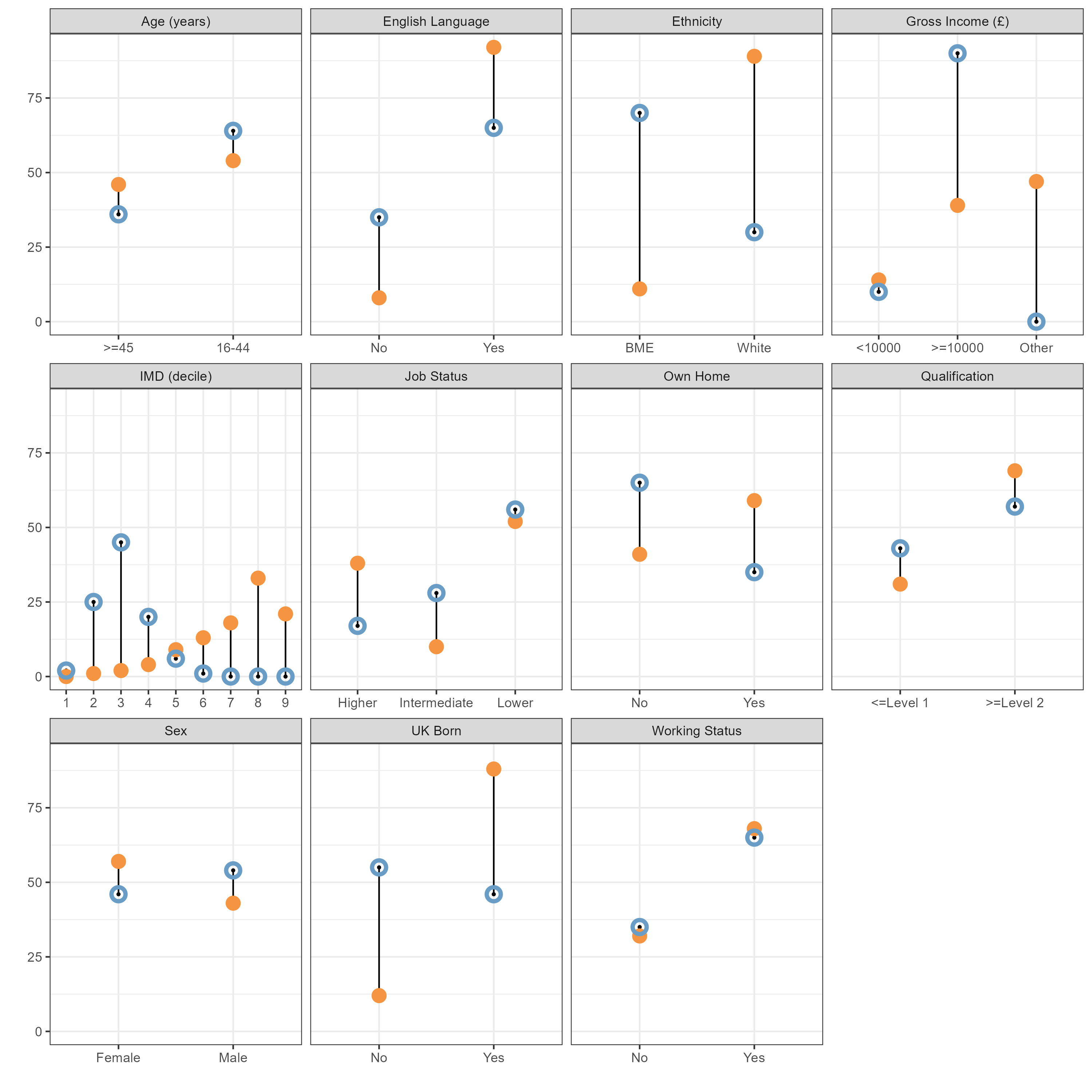

Supplement: Supplementary file 1 — Supplementary Material 1. [file 12889_2025_26067_MOESM1_ESM.zip › dumbbell_plot_literacy.png]

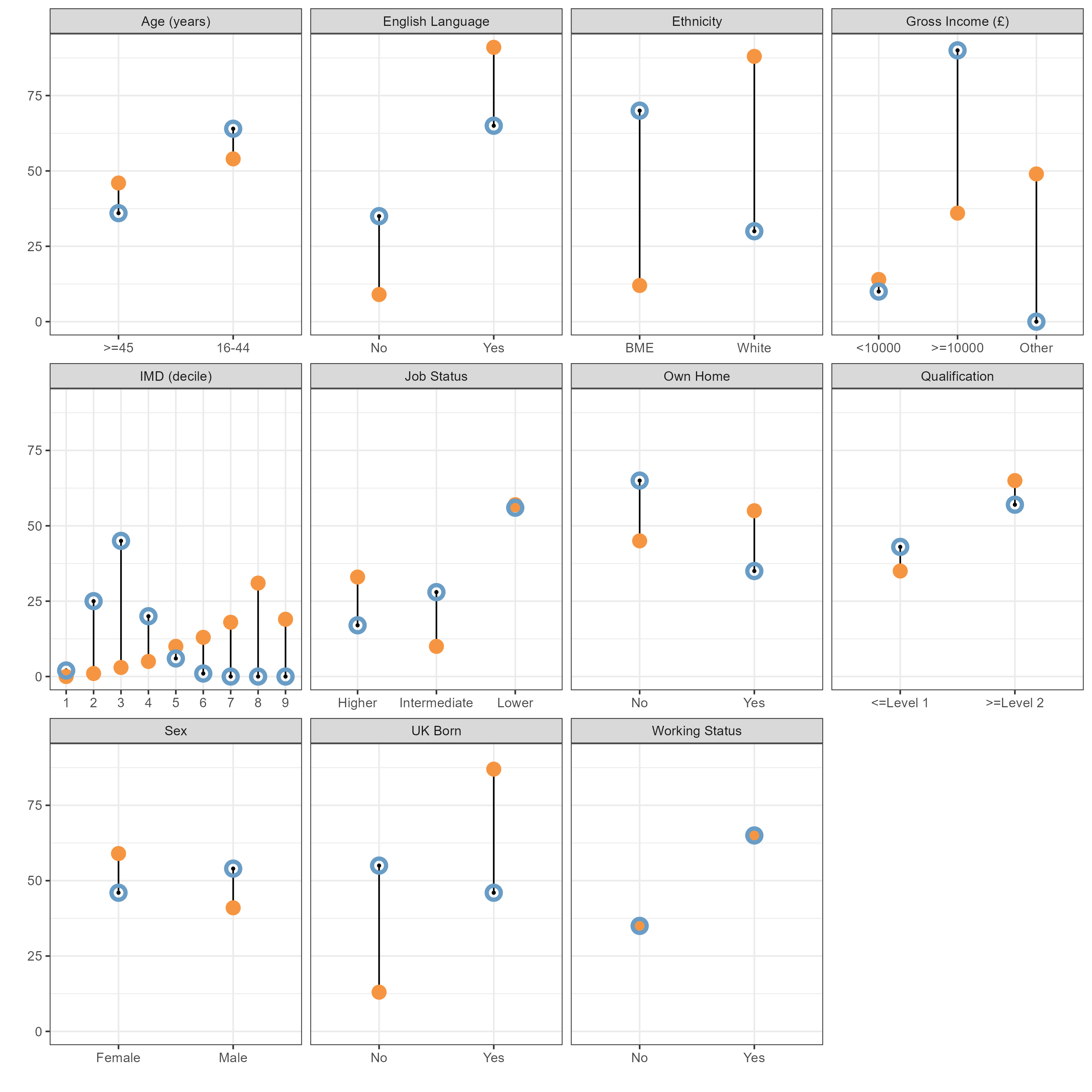

Supplement: Supplementary file 1 — Supplementary Material 1. [file 12889_2025_26067_MOESM1_ESM.zip › dumbbell_plot_numeracy.png]

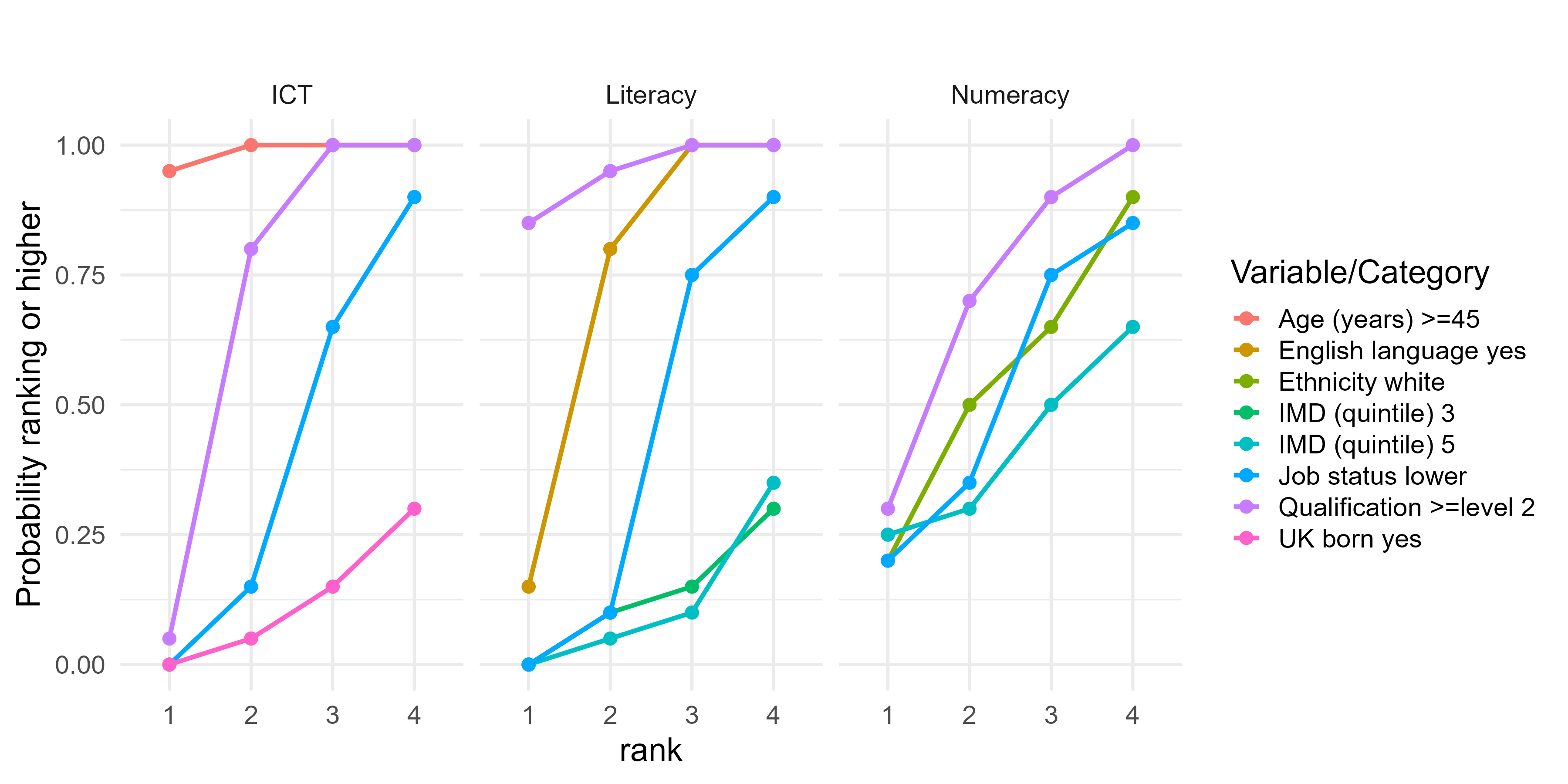

Supplement: Supplementary file 1 — Supplementary Material 1. [file 12889_2025_26067_MOESM1_ESM.zip › ame_sucra_group_plot.png]

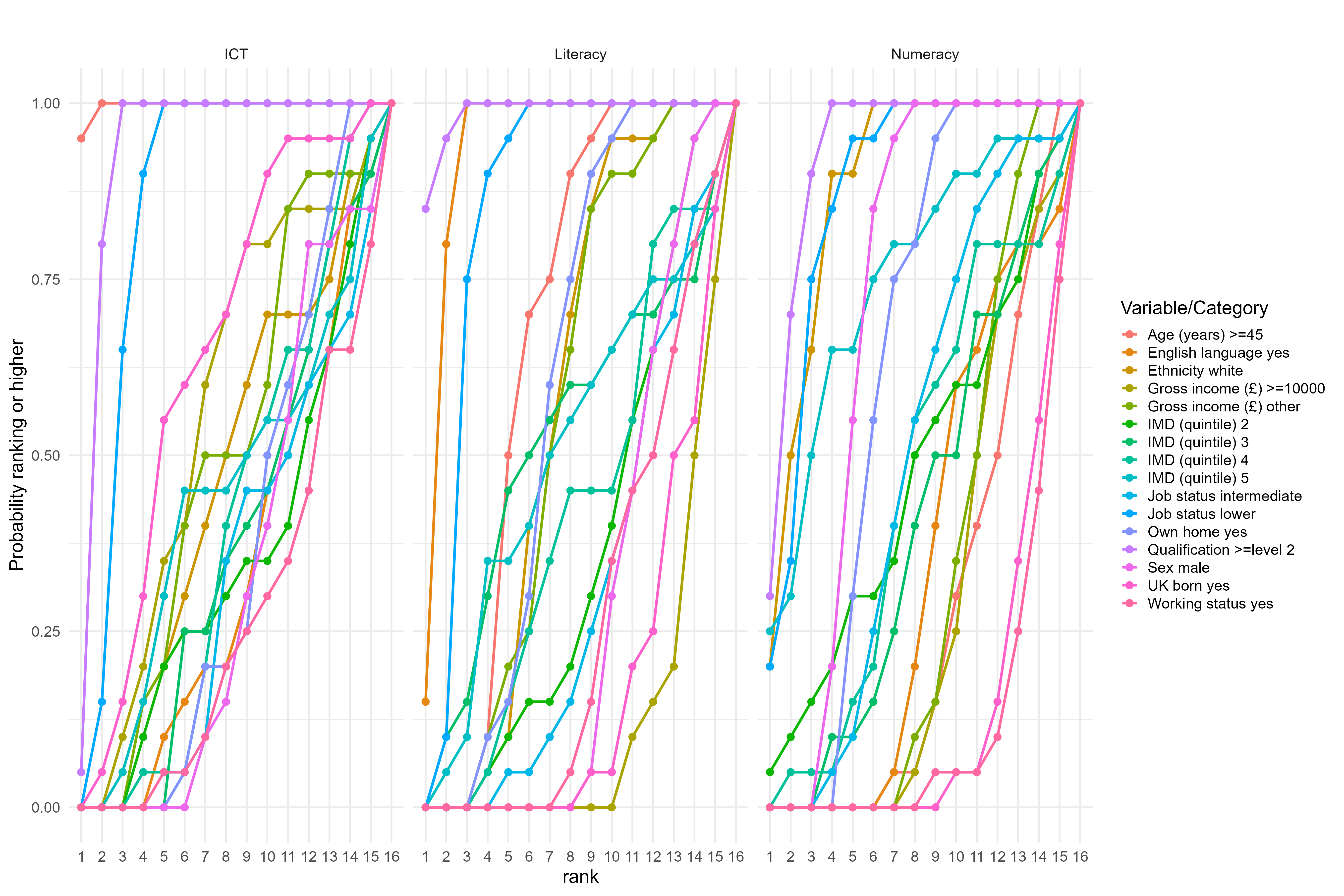

Supplement: Supplementary file 1 — Supplementary Material 1. [file 12889_2025_26067_MOESM1_ESM.zip › gg_cumrank_complete.png]

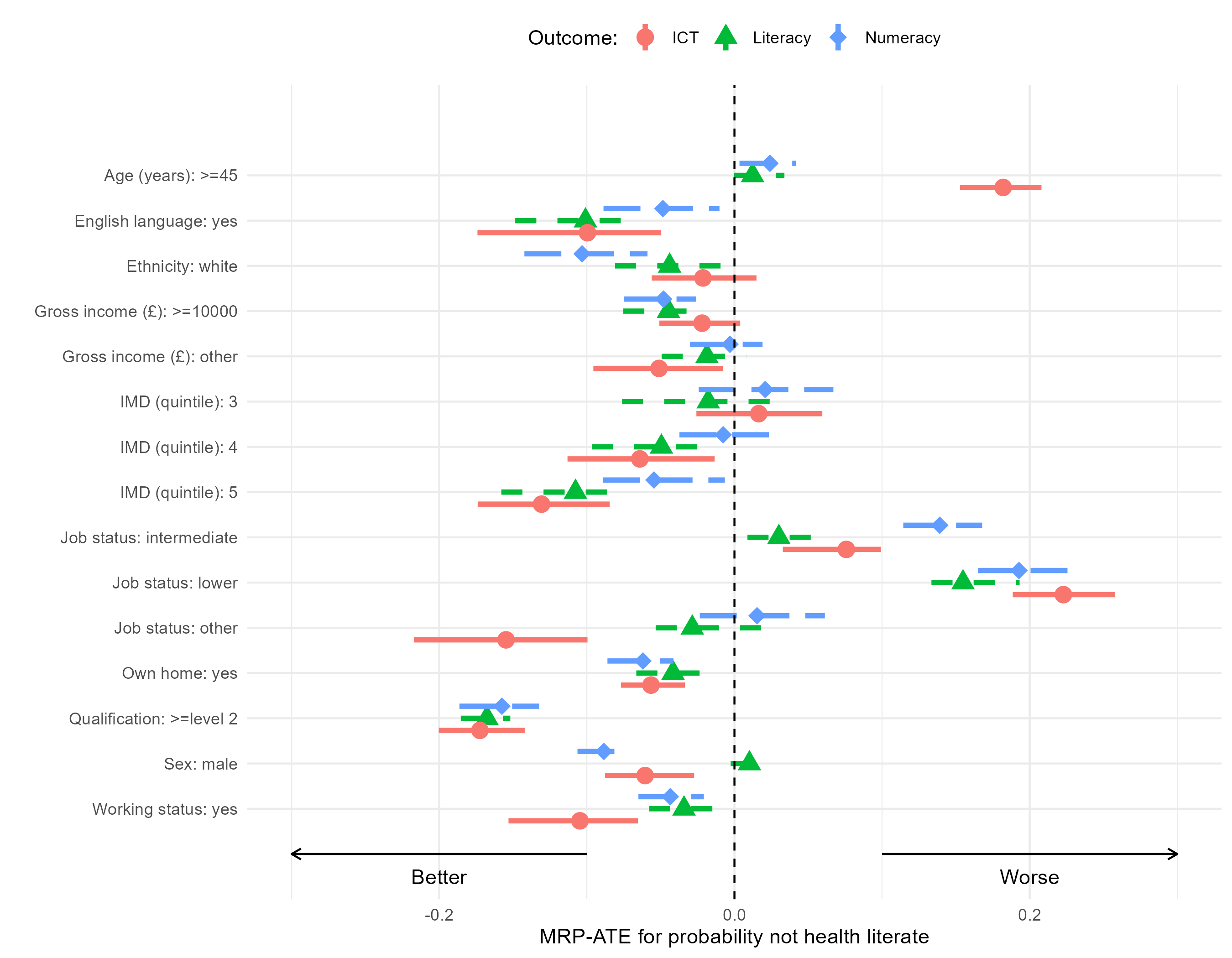

Supplement: Supplementary file 1 — Supplementary Material 1. [file 12889_2025_26067_MOESM1_ESM.zip › ame_forest_group_plot_2003.png]

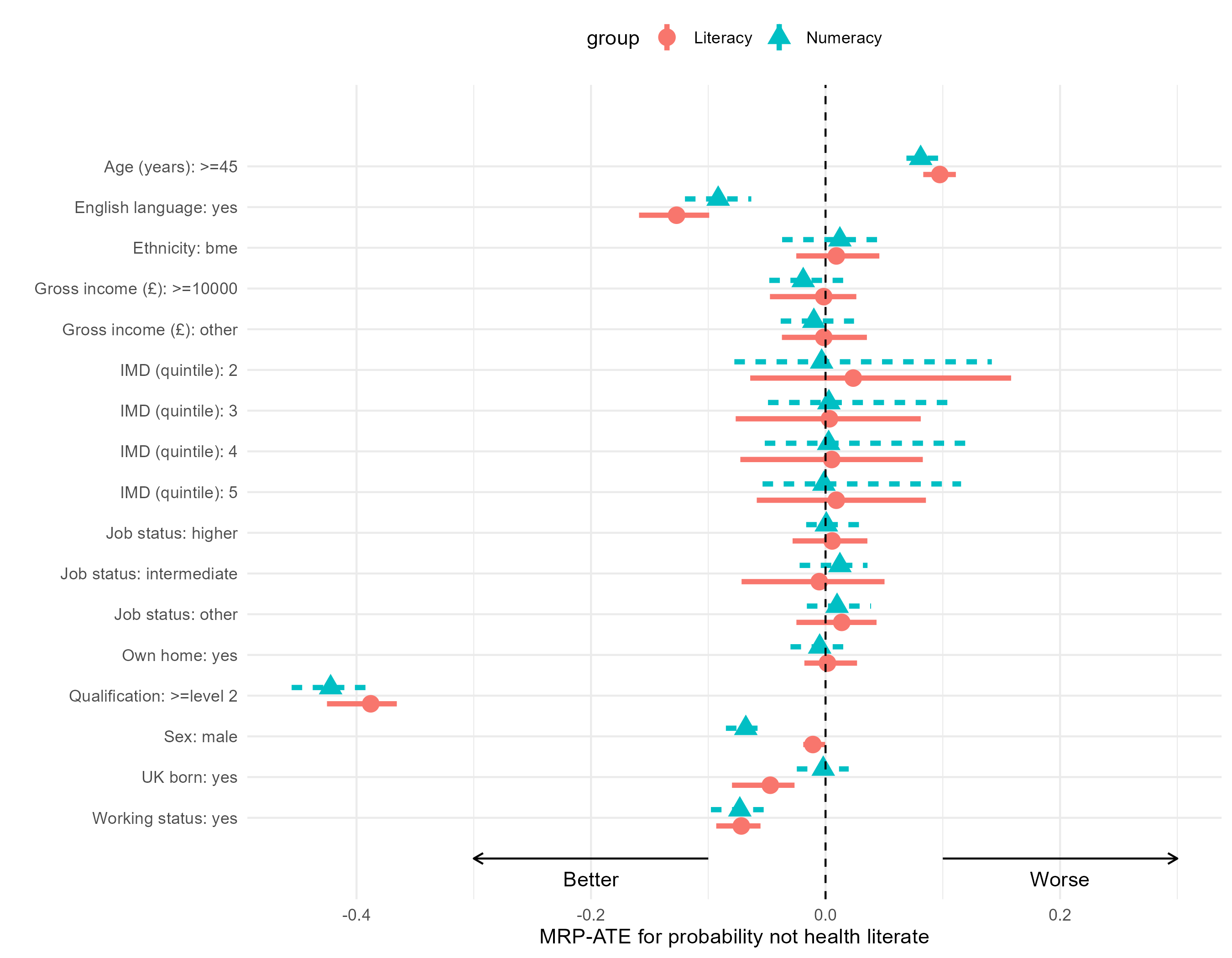

Supplement: Supplementary file 1 — Supplementary Material 1. [file 12889_2025_26067_MOESM1_ESM.zip › ame_forest_group_plot_piaac_imp.png]
